# 1419. Evaluation of a vaporized hydrogen peroxide sterilization technology for duodenoscopes

**DOI:** 10.1093/ofid/ofad500.1256

**Published:** 2023-11-27

**Authors:** Martin M Varghese, Jennifer Cadnum, Samir Memic, Maria M Torres-Teran, William Rutala, Curtis Donskey

**Affiliations:** The Cleveland VA Medical Research and Education Foundation, Cleveland, Ohio; Northeast Ohio VA Medical Center, Cleveland, Ohio; Northeast Ohio VA Healthcare System, Cleveland, Ohio; Northeast Ohio VA Medical Center, Cleveland, Ohio; University of North Carolina, Chapel hill, NC; Cleveland VA Hospital, Cleveland, Ohio

## Abstract

**Background:**

Current methods for reprocessing duodenoscopes, including double high-level disinfection, are not effective in eliminating bacterial contamination. Thus, there is a need to develop effective and safe sterilization technologies for duodenoscopes.

**Methods:**

We examined the effectiveness of a new hydrogen peroxide gas plasma sterilizer designed for terminal sterilization of flexible endoscopes. The technology directs vaporized hydrogen peroxide into all internal endoscope channels with a short exposure time and includes a sterilization container that facilitates sterile storage after processing. Test organisms included *Bacillus atrophaeus* and *Clostridioides difficile* spores, *Escherichia coli*, *Enterococcus faecium*, and *Candida auris* inoculated on steel wires or dispersed throughout the internal channels with 5% fetal calf serum.

**Results:**

The technology reduced >10^8^ colony-forming units (CFU) of the vegetative organisms to undetectable levels. *B. atrophaeus* and *C. difficile* spores on steel wires were eliminated by the technology ( >6 log_10_ CFU reduction) and spores disperse throughout the channels were reduced by >5 log_10_ CFU. No damage to the endoscopes was observed.

**Conclusion:**

The sterilization technology was highly effective in reducing contamination of duodenoscopes in laboratory testing. Our results support further investigation of this technology in reducing contamination of in-use endoscopes.

**Disclosures:**

**William Rutala, MS, MPH, PhD**, Ideate Medical: Advisor/Consultant|Ideate Medical: Stocks/Bonds|Kinnos: Advisor/Consultant|Kinnos: Stocks/Bonds|PDI: Advisor/Consultant|PDI: Honoraria

